# The First Issue: Innovations in Teaching, Assessment, and Developing a Robust Educational Pipeline in Clinical Neuroscience

**DOI:** 10.1212/NE9.0000000000200009

**Published:** 2022-10-19

**Authors:** Roy Strowd

**Affiliations:** From the Department of Neurology, Wake Forest University School of Medicine, Winston Salem, NC.

The first issue of *Neurology*® *Education* is here. The journal publishes original research articles, curriculum innovations, and evidence-based teaching in neurologic education across all stages of training. Since launching in April, the journal has received numerous important papers on educational scholarship in the clinical neurosciences. Articles are published online continuously allowing rapid dissemination for authors. To facilitate reading, articles are assembled periodically into issues and are arranged thematically to engage readers and organize content around common themes. In this issue, readers will find articles arranged thematically in 3 areas: (1) novel approaches to teaching clinical neurology with a focus on curricular lessons learned from the coronavirus disease 2019 (COVID-19) pandemic, (2) narrative evaluations and how a faculty development program can improve narrative assessment of medical students, and (3) innovative curricula that enhance the pipeline of students pursuing careers in clinical neuroscience.

## Innovations in Teaching

The COVID-19 pandemic catalyzed widespread adoption of online and eLearning initiatives. Lessons learned from these past 2 years are now influencing the next steps in how clinicians are trained in clinical neuroscience. In the first article in the issue, Albin et al.^1^ address a fundamental question in simulation education: are high-fidelity manikins equivalent in educational value to live actors? They performed a pilot study exploring neurology resident performance on a simulated neurologic emergency, and they show similar improvements in knowledge and confidence with the use of standardized patients and manikins. They discuss how manikins can overcome barriers of cost and lack of standardization, and contrast these with the benefits of direct feedback to learners with standardized patients. Ultimately, high quality debriefing may have been a key ingredient in both settings.

Small residency and subspecialty fellowship programs in neurology, neurosurgery, and psychiatry face unique challenges because of limited resources and the need to standardize curricula. Program directors struggle to deliver comprehensive didactics or create networking for 1 or 2 fellows. The second article in this issue, by Peters et al.,^2^ describes the implementation of an interinstitutional online seminar series in neuroimmunology. The program brought together fellows from across the nation to learn neuroimmunology. The lessons learned by this group include how to harness excitement for interinstitutional learning, how to deliver standardized teaching, and how collaboration between fellows can actually be achieved online. The article is relevant to fellowship program directors in the clinical neurosciences who are struggling with similar challenges.

## Narrative Assessment in Clinical Neuroscience

The next article in this issue addresses a key aspect of evaluation in clinical training: narrative assessment. According to the Accreditation Council for Graduate Medical Education Milestone Project, meaningful narrative assessment is a vital component of direct observation and is often the most helpful information for program directors.^3^ Clinical competency committees (CCCs) rely on meaningful narrative comments when making milestone determinations. In the study by Mooney et al.,^4^ a faculty development program informed by the theory of deliberate practice significantly improved the quality of narrative assessments. This study provides a framework for other clerkships, residencies, and CCCs seeking to improve narrative feedback.

## Bringing Awe and Excitement Back Into Training

Educating and evaluating the future generation of clinicians in the neurosciences could not be more important today. The global burden of neurologic disease is expanding and will continue to outpace the supply of neurologists, neurosurgeons, and psychiatrists.^5-7^ In the past half decade, the number of undergraduate institutions with neuroscience majors has increased by 41%.^8^ However, graduate training is not keeping pace. Today, only 2.5% of medical students select a career in neurology; 1% select neurosurgery; and 6% select psychiatry.^9^ Many have suggested that the complexity of the nervous system, the lack of engaging neuroscience teaching, and a failure to link basic neuroscience to clinical neurology early in training result in neurophobia and contribute to the attrition away from careers in clinical neuroscience.^10^ Much has been written about neurophobia. Although arguably, little success has been made since its description in 1994.^11^ In many ways, this may be the result of a misdiagnosis. Many fields in medicine are complex. Linking basic science concepts to clinical medicine is an aspiration not just for neurology but for many fields that are taught in medical school. Neurology is not a mandatory clerkship at some institutions, but the same is true for wildly popular fields such as anesthesiology and radiology.

In many respects, what lies at the root of neurophobia may be a loss of the excitement and amazement for the brain. Do you remember the first time you were truly fascinated by the nervous system? For so many clinicians, the initial draw into the field was an awe with the brain, mind, or psyche. Undergraduate students majoring in neuroscience often develop an unquenchable thirst for this fascination and frontier. The transition to learning clinical pathology has a tendency to replace excitement about the brain with a disillusionment for neurologic disease? Several articles in this issue explore curricula that bring fascination and excitement back into clinical neuroscience at different stages of the transition from undergraduate to medical school.

In the first of these articles, Minen et al.^12^ present results of a large survey of 140 basic neuroscience faculty instructors. The authors sought to identify opportunities to bring clinical neurology into undergraduate neuroscience instruction. The article provides a roadmap for integrating the excitement of clinical neurology early into undergraduate training. In the next article, Fuentes et al.^13^ present a longitudinal PreDoc program that links undergraduate institutions with clinical training programs. Students peer into the lives of neuroscience clinicians and identify the passions and sense of belonging that pursuing a career in neurology brings. This program also addresses diversity and inclusion describing how unique barriers can be overcome for students from marginalized backgrounds. In the final article, Sanderson et al.^14^ bring the excitement of procedural neurology to second-year medical students and link the learning of basic neuroscience with the fun of procedural medicine.

This issue appeals to educators at all levels of neurologic and neuroscience training and reflects the journals' mission to publish high-quality education research relevant to all fields, all professions, and all learners in the clinical neurosciences. We welcome your submissions. Happy reading.
